# Full Mouth Rehabilitation Using the Twin Stage Procedure in a Patient with Amelogenesis Imperfecta: A Case Report

**DOI:** 10.7759/cureus.25512

**Published:** 2022-05-31

**Authors:** Ashish Dadarwal, Jyoti Paliwal, Vineet Sharma, Surendra Jaswal, Rajkumar Meena

**Affiliations:** 1 Prosthodontics, Rajasthan University of Health Sciences (RUHS) College of Dental Sciences, Jaipur, IND; 2 Oral Medicine and Radiology, Pacific Dental College and Hospital, Udaipur, IND; 3 Periodontology, Rajasthan University of Health Sciences (RUHS) College of Dental Sciences, Jaipur, IND

**Keywords:** amelogenesis imperfecta, zirconia, twin stage procedure, hobo, full mouth rehabilitation

## Abstract

Amelogenesis imperfecta (AI) refers to a group of inherited odontological disorders that alter enamel formation. The AI variant is based on the primary enamel defect, classified as hypoplastic type I, hypo maturation type II, hypo calcification type III, and hypo maturation type IV. AI is commonly linked with the loss of the normal occlusal plane, the loss of the vertical dimension of occlusion (VDO), and impaired functions and esthetics. This case report describes the Hobo and Takayama twin-stage procedure for the rehabilitation of a patient with hypoplastic AI.

## Introduction

AI is a group of inherited odontological disorders that disrupt enamel formation. Only epithelial derivatives exhibit the disorder. AI does not affect the mesenchymal derivatives of the developed tooth bud; hence dentine, cement, and pulp remain normal. AI typically affects both deciduous and permanent teeth [1]. All or a subset of the teeth in an arch can be affected. AI clinical features include impacted teeth, follicular cysts, open interproximal contacts, excessive wear, and open-occlusal skeletal relations [2,3]. AI is believed to occur at a rate of 1:700 to 1:14,000 in the overall population [4]. AI is more prevalent in isolated and stable population zones, accounting for the wide frequency range.

Normal enamel development occurs in three distinct phases in AI: the formative phase, the calcification phase, and the maturation phase. The organic enamel matrix is synthesized during the formative phase, mineralized during the mineralization phase, and the enamel crystals mature during the calcification and maturation phases [5].

The AI variation is based on the primary enamel defect, classified as hypoplastic type I, hypo maturation type II, hypo calcification type III, and hypo maturation type IV. Fourteen AI subtypes were identified by inheritance pattern, histological assessment, and radiographic and clinical appearance [5,6]. Autosomal or X-linked, dominant, or recessive genes may cause AI. If we look at family history and build a pedigree, we will discover whether a heritage pattern is autosomal or X-connected [7,8]. 

The twin-stage technique developed by Hobo and Takayama develops anterior guidance to create a predetermined and harmonious posterior disocclusion with the condylar path [9]. This clinical case report discusses the Hobo and Takayama twin-stage technique for rehabilitation of a patient with AI, decreased vertical dimension of occlusion (VDO), and esthetically and functionally compromised dentition.

## Case presentation

**Figure 1:**
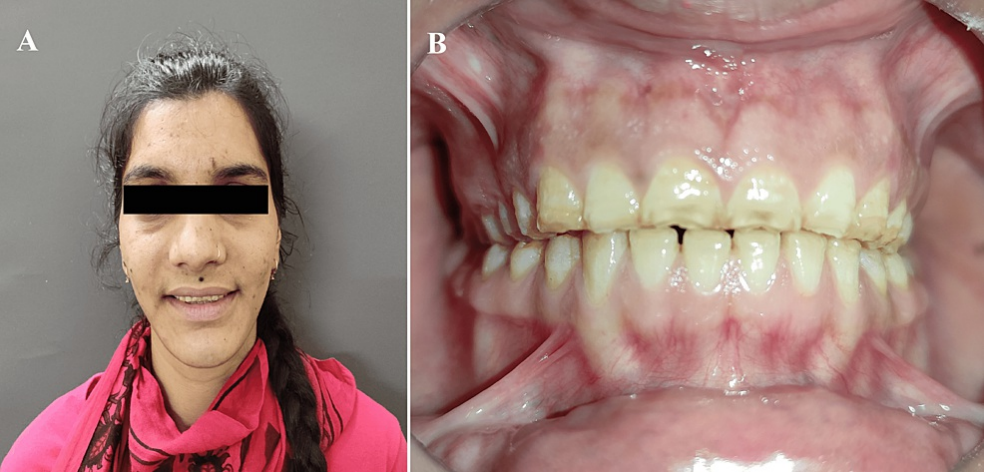
Pre-operative images (A) Extraoral View; (B) Intraoral View

A 24-year-old female presented to the Prosthodontics Department at the Rajasthan University of Health Sciences (RUHS) College of Dental Sciences in Jaipur, India, with the chief complaint of worn-out flat teeth with compromised esthetics, generalized sensitivity to hot and cold, and difficulty chewing (Figure 1). There was no significant medical history. Her elder brother was also suffering from AI hypoplastic type. The extraoral examination did not reveal facial asymmetry or muscle tenderness. Movements of the mandible also appeared to be within normal limits. There were no symptoms of temporomandibular joint dysfunction or myofascial pain dysfunction. Her physical growth was within normal limits, and she was average in stature, appearance, height, and weight for her age group.

**Figure 2:**
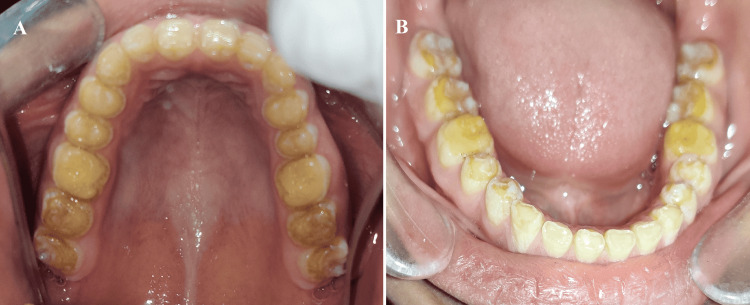
Intraoral occlusal view (A) Maxilla; (B) Mandible

Intra-oral examination revealed generalized attrition, a yellow to brownish color, and smaller teeth (Figure 2). A 5-millimeter (mm) freeway space was evaluated. On radiographic examination, there was an absence of any abnormality in the pulp chambers and pulp horns of the teeth. In addition, there was no peri-apical radiolucency or radio-opacity.

The patient was referred to the Department of Oral Medicine to rule out developmental disorders or syndromes. Upon clinical, radiographic, and family history evaluation, a diagnosis of AI hypoplastic type was finally made for the patient. Clinical findings and freeway space analysis led to reconstructing the dentition with a 2 mm raised VDO.

When restoring a short clinical crown, a subgingival margin can be used to increase crown length. However, deep subgingival margins encroach on biologic width and are hence undesirable. Because the sulcus depth for each anterior tooth, in this case, was 2 mm, it was planned to increase the crown height by clinical crown lengthening with osseous recontouring to achieve a consistent biological width of 3 mm for each tooth.

The treatment options explained to the patient were full-mouth bonded overlay crown restorations or full-mouth crown restorations. The patient was finally rehabilitated through full-mouth zirconia crown restorations using Hobo's twin-stage technique.

Clinical procedure

**Figure 3:**
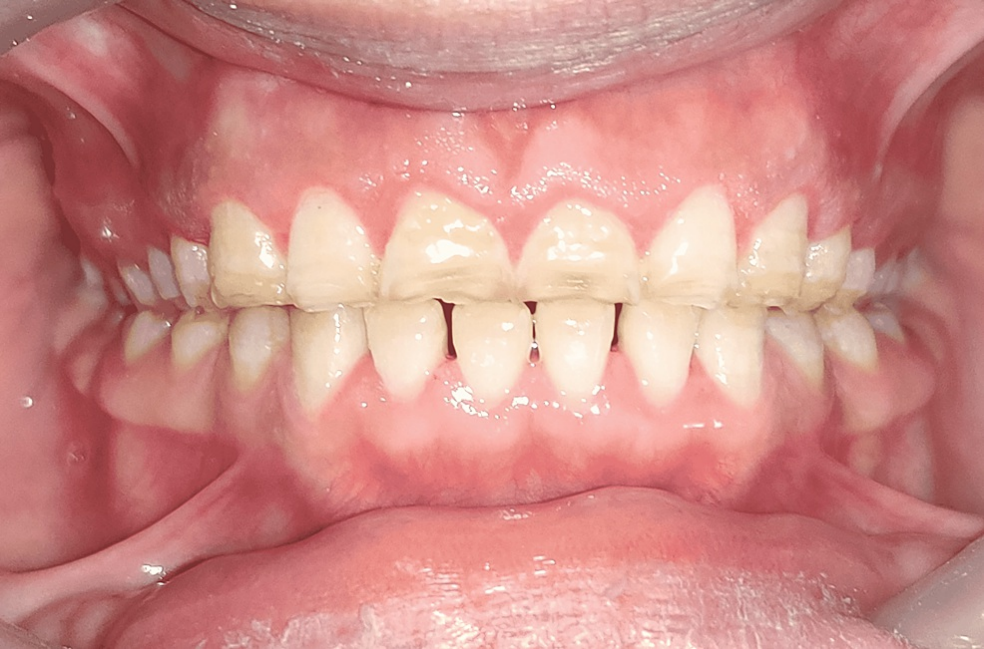
Intraoral view after clinical crown lengthening

The patient was referred to the department of periodontics for clinical crown lengthening (Figure 3). After clinical crown lengthening, diagnostic impressions were made in the irreversible hydrocolloid impression material (Zelgan; Dentsply, New Delhi, India) to get diagnostic casts.

**Figure 4:**
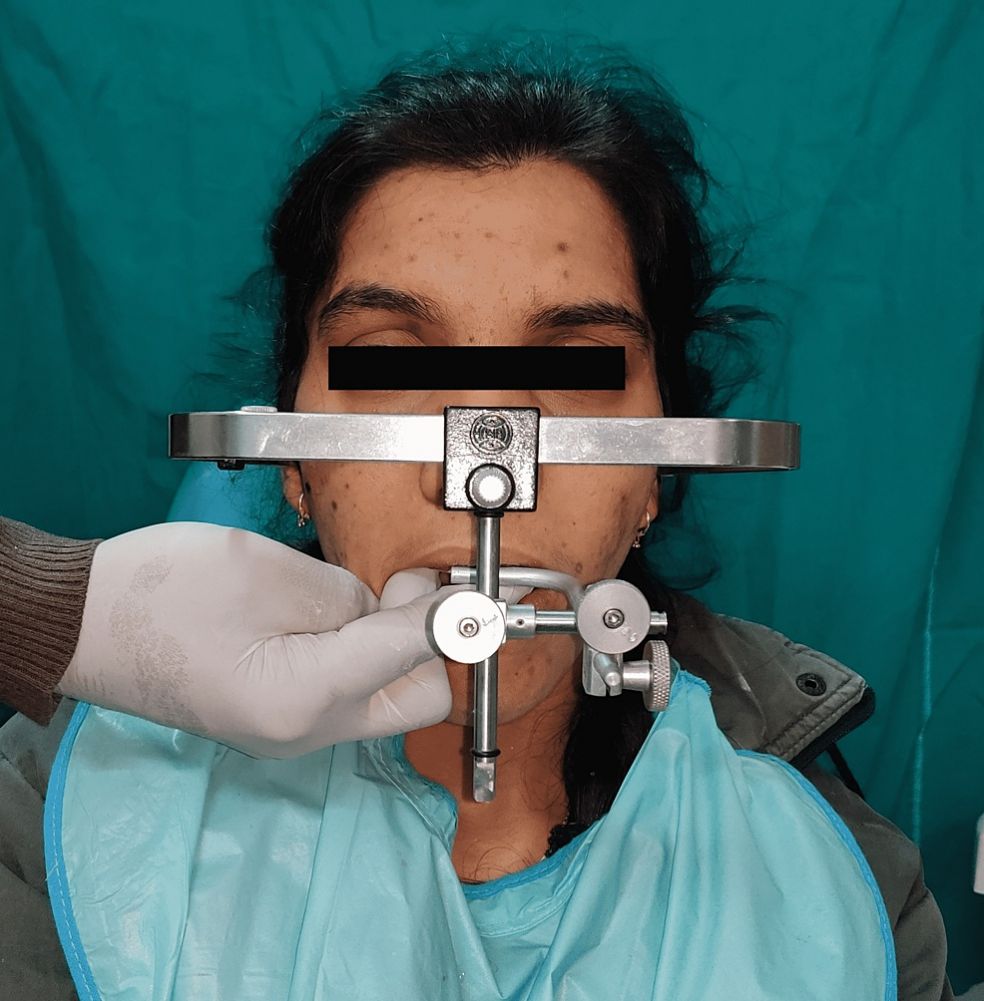
Face bow record

**Figure 5:**
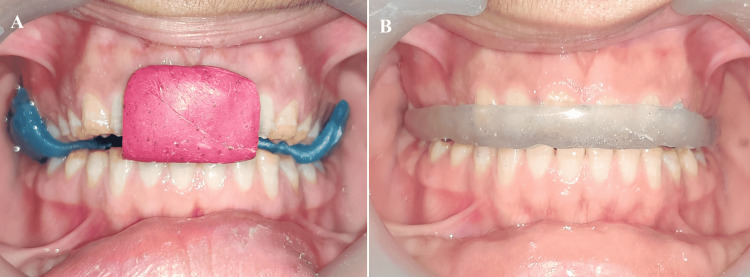
The patient's adaptation to the altered VDO (A) Centric record at 2 mm raised VDO; (B) Occlusal splint at 2 mm raised VDO VDO: Vertical dimension of occlusion

A face bow record was made using a maxillary cast on a semi-adjustable articulator (Hanau™ Wide-Vue; Whip Mix Corporation, Louisville, USA) (Figure 4). A centric record was obtained using a custom-made jig in the anterior teeth. The inter-occlusal recording material (Alu Wax; I-Med Dental Solutions, Vadodara, India) was placed on the posterior teeth. The mandibular cast and the centric record were mounted on the articulator. After that, an occlusal splint was fabricated at the raised VDO to evaluate the patient's adaptation to the altered VDO (Figure 5).

**Figure 6:**
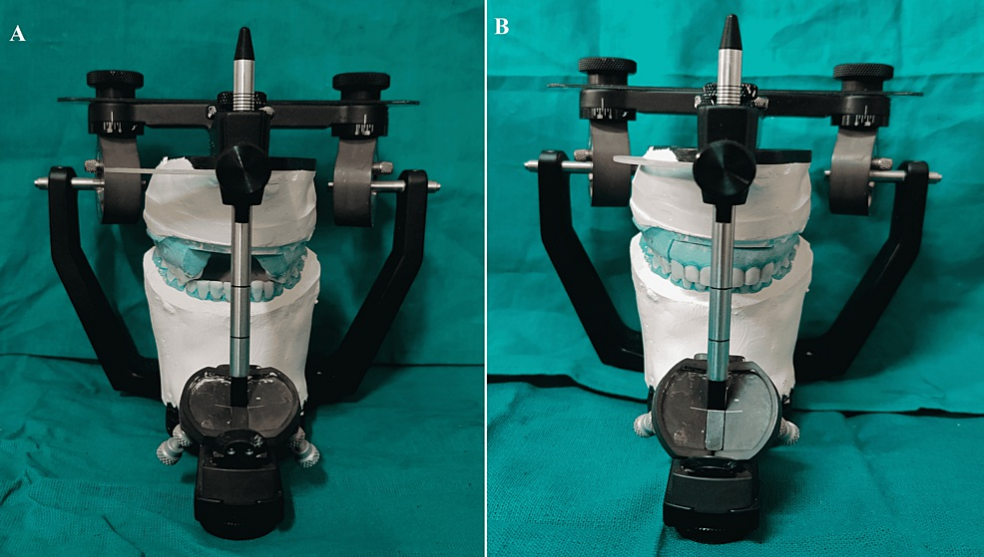
Diagnostic wax-up (A) Condition 1; (B) Condition 2

To confirm, the patient was kept under observation for six weeks. After that, a diagnostic wax-up was done. Without the anterior segment of the maxillary cast in place, a diagnostic wax-up of the full-mouth restoration was done at the increased VDO for posterior teeth. The condylar and incisal guidance was set to Condition 1 to produce standard effective cusp angles. At this point, the diagnostic wax-up was balanced in the protrusive and lateral excursion. After reassembling the anterior section of the cast, the condylar and incisal guidance was set to Condition 2, and the wax-up produced posterior disocclusion (Figure 6). A putty index of diagnostic wax-up was then made to fabricate provisional crowns by an indirect technique. Provisional crowns were made in three segments (one anterior and two posteriors) and provisionally cemented over the unprepared teeth of both arches to see esthetic changes before tooth preparation. Provisionals also act as a stop to allow for uniform occlusion reduction of the opposing arch.

**Figure 7:**
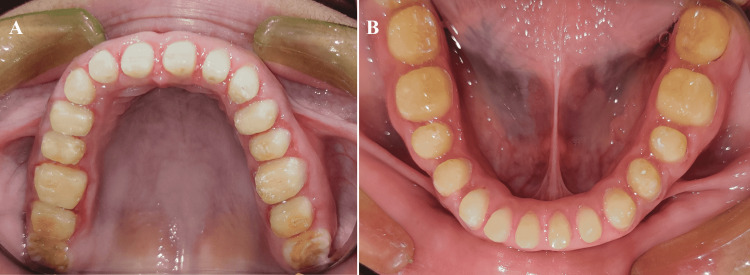
Teeth preparation (A) Maxilla; (B) Mandible

**Figure 8:**
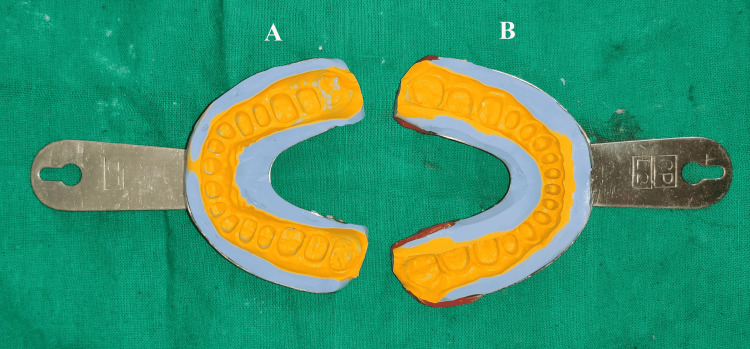
Master impression with addition silicon (A) Maxillary arch; (B) Mandibular arch

After obtaining esthetic clearance from the patient, all teeth were prepared in one appointment with a minimal occlusion reduction, as recommended for patients with an increased VDO (Figure 7). After proper gingival tissue retraction, master impressions of maxillary and mandibular prepared teeth were made with elastomeric impression material (GC Flexceed; GC India, Telangana, India) using the dual step putty wash technique (Figure 8).

**Figure 9:**
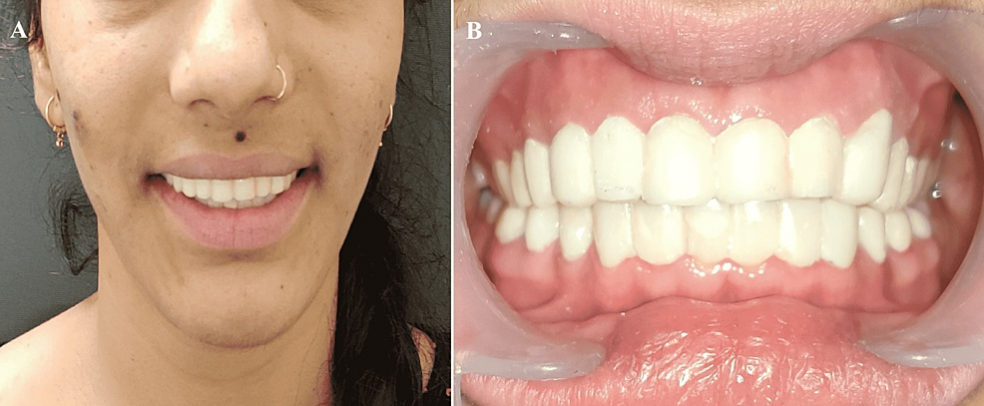
Provisional crowns in situ (A) Extraoral; (B) Intraoral View

Provisional crowns were relined with self-cure acrylic resin (SC-10; PYRAX, Roorkee, India) to accommodate the space left after tooth preparation (Figure 9). Interferences in centric as well as eccentric movements were eliminated. In the centric relation (CR), maximum intercuspation was achieved, and the protrusion obtained posterior disclusion. The patient's adaptation was observed for six weeks. The esthetics and functional harmony of the patient was assessed.

**Figure 10:**
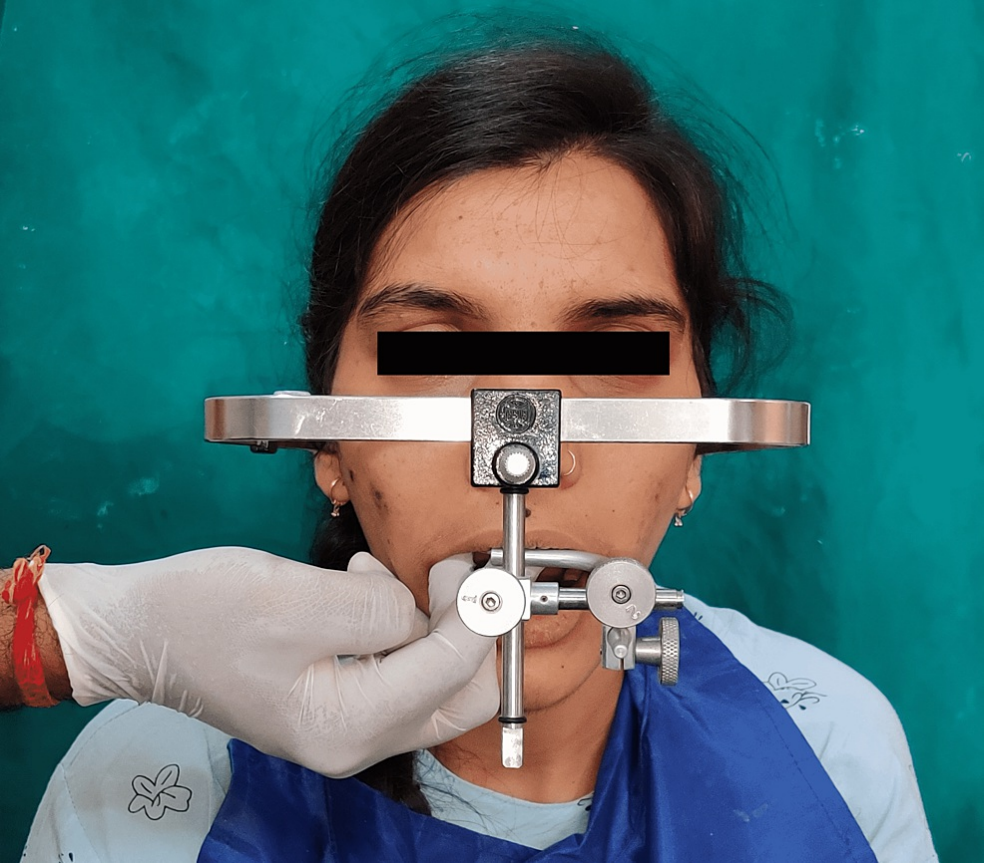
New face bow record after tooth preparation

**Figure 11:**
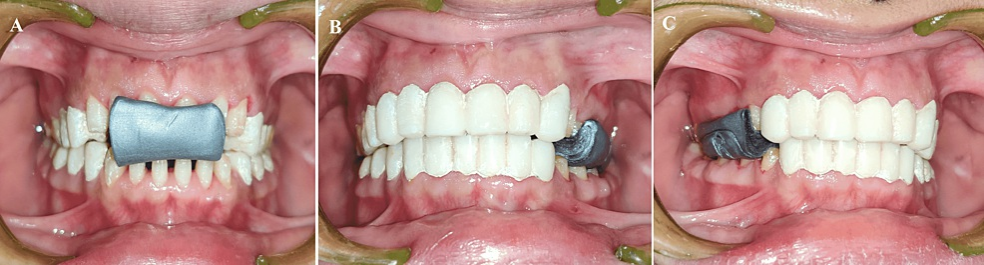
Three segmental inter-occlusal records to transfer VDO and CR (A) Anterior; (B) Left Posterior; (C) Right Posterior VDO: Vertical dimension of occlusion; CR: Centric relation

The maxillary cast was mounted on a semi-adjustable articulator with the face-bow (Figure 10). First, provisional crowns were removed from the left posterior maxillary and mandibular areas to transfer the VDO and CR. The provisional crowns of the right and anterior maxillary and mandibular regions, on the other hand, served as a stop. Afterward, inter-occlusal recording material (Alu Wax; I-Med Dental Solutions, Vadodara, India) was placed between the prepared teeth on the left sides. Likewise, provisionals were removed from the right side but kept in the left and anterior regions of both arches. Next, an inter-occlusal record was placed between the prepared teeth on the right side. A similar technique was followed in the anterior area (Figure 11). Finally, the three segmental inter-occlusal records obtained were used to mount the mandibular cast. A non-eugenol temporary cement (NETC; Meta Biomed, Colmar, USA) was used to reposition the provisional crowns.

**Figure 12:**
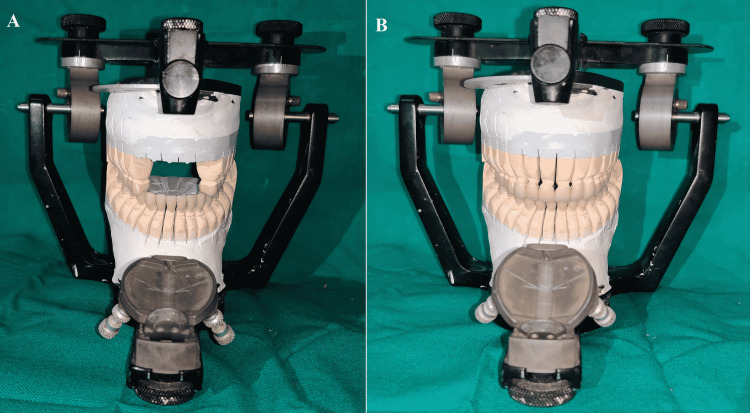
(A) Condition 1; (B) Condition 2

**Figure 13:**
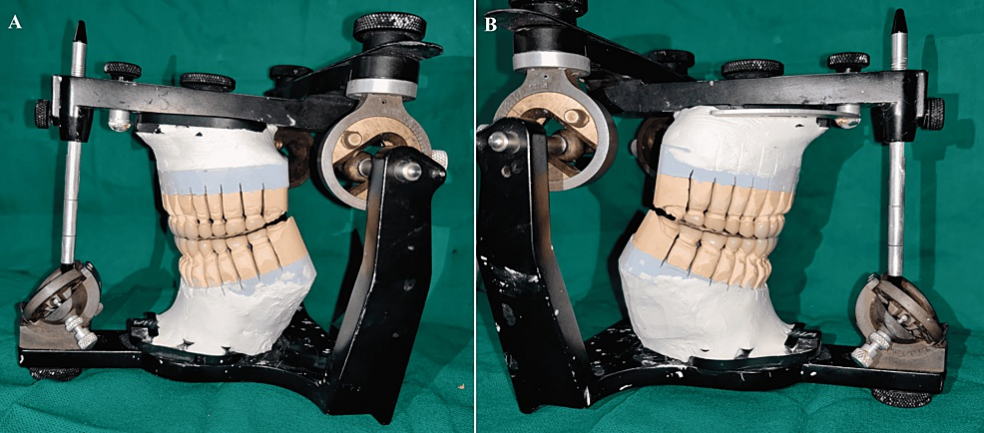
Disocclusion in protrusion (A) Left view; (B) Right view

**Figure 14:**
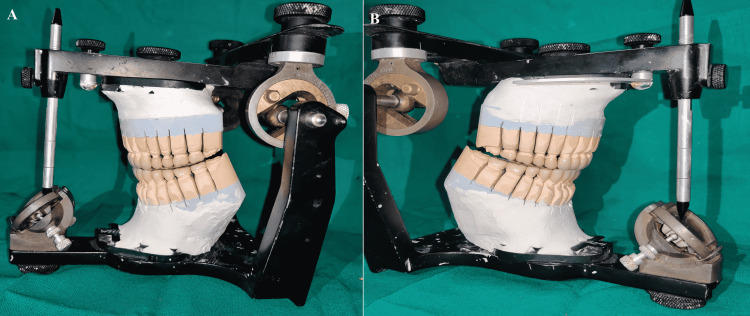
Disocclusion in left working (A) Left side; (B) Right side

**Figure 15:**
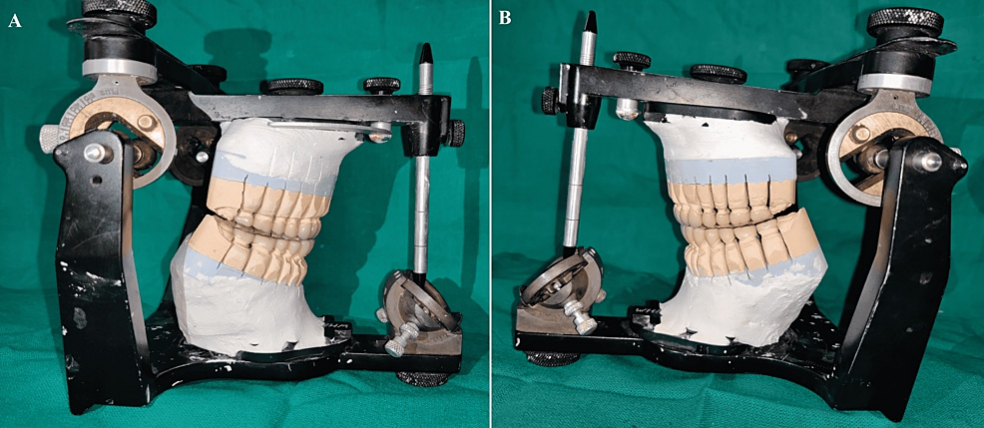
Disocclusion in right working (A) Right side; (B) Left side

**Figure 16:**
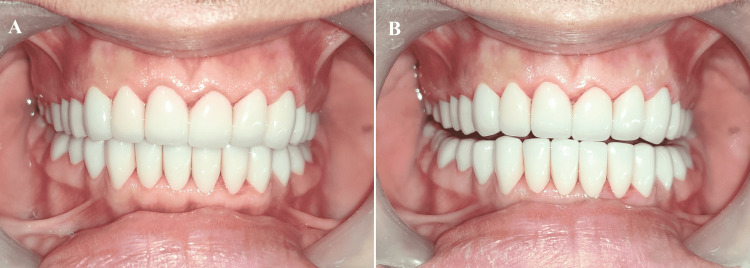
Final prostheses (A) Final prostheses in situ; (B) Posterior disocclusion in protrusion

**Figure 17:**
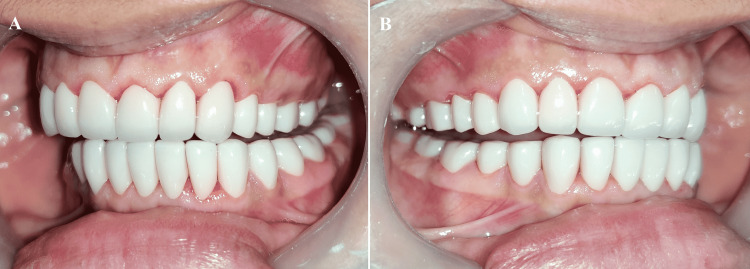
Posterior disocclusion in the left eccentric movement (A) Left side; (B) Right side

**Figure 18:**
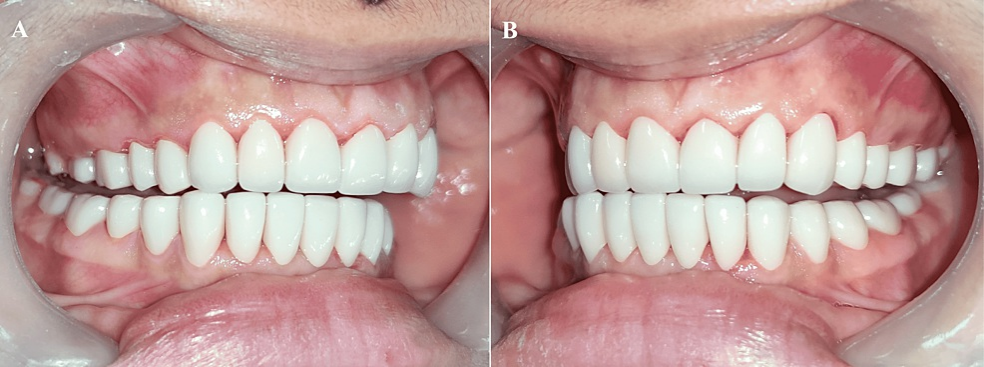
Posterior disocclusion in the right eccentric movement (A) Right side; (B) Left side

**Figure 19:**
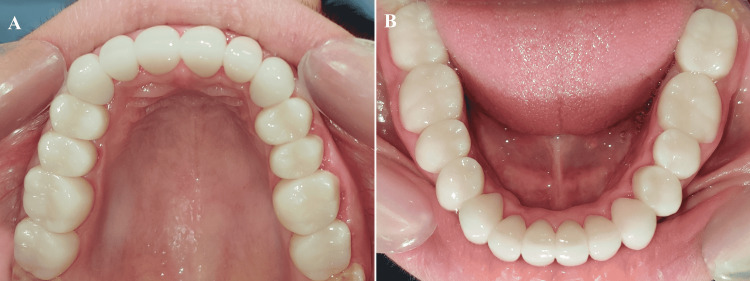
Occlusal view (A) Maxillary Arch; (B) Mandibular Arch

**Figure 20:**
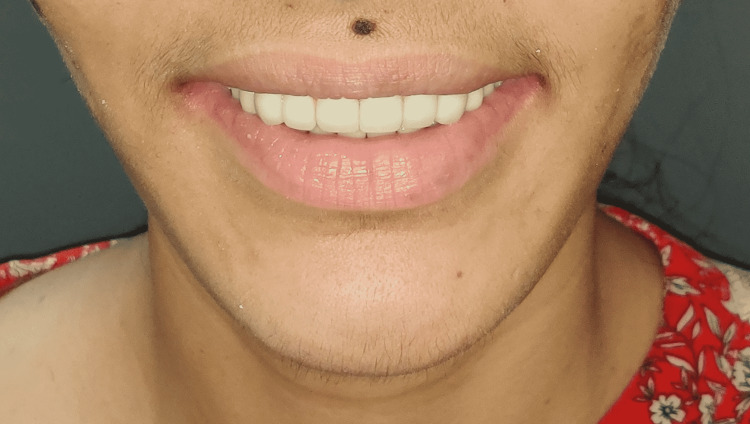
Smile of satisfaction (Final restoration in situ)

Maxillary and mandibular casts were scanned and transferred to CAD software (DentalCAD; Exocad GmbH, Darmstadt, Germany) to create three-dimensional virtual models. The margins were marked manually, and an appropriate insertion path was determined. The CAD software was used to design complete coverage restorations and adjust them over virtual models. Following the design phase, milling was carried out by a five-axis CNC machine (ME-300HP; TDS Biotechnology Co. Ltd., LMT, Newtown, USA). The final restorations were cemented with dual-cure resin cement (Fusion Ultra D/C; Prevest Denpro Limited, Jammu, India). The patient was provided a mutually protected occlusion scheme (Figures 12-20). The definitive restorations were made with high translucent monolithic zirconia (BruxZir® Esthetic Zirconia; Glidewell Dental, California, USA) crowns for maxillary anterior and high-strength monolithic zirconia crowns (BruxZir® Full-Strength Zirconia; Glidewell Dental, California, USA) for the remaining teeth, exhibiting a vital and natural appearance with the appropriate contour and shade. The patient was given oral hygiene instructions that emphasized dental floss and thorough brushing, and a six-week follow-up was scheduled.

## Discussion

The clinical manifestations of AI differ depending on the type. The hypoplastic type has well-mineralized enamel, but its quantity is reduced [5,6]. The VDO was raised by 2 mm since the patient had a reduced VDO and a 5 mm freeway space [10,11]. As anterior teeth wear away, it facilitates the loss of anterior guidance that protects the posterior teeth during excursive movements. The collapse of the posterior dentition results in the loss of a normal occlusal plane and a reduction in vertical dimension [12].

The full-mouth rehabilitation (FMR) process is designed to restore the masticatory process's health, esthetics, and efficiency [13]. The most critical requirements are healthy temporomandibular joint (TMJ), harmonious anterior guidance, and non-interfering posteriors [14]. These three factors are intertwined, and any imbalance among them will impact the stomatognathic system. The diagnostic wax-up should always precede the treatment to decide on the appearance, remove occlusal interferences, and predict the required amount of tooth preparation. It is necessary to do a diagnostic wax-up to determine the desired esthetics, tooth contours, position of teeth, and occlusal plane. Additionally, it facilitates the fabrication of provisional restorations, which saves time.

Following centric relation, anterior guidance plays a critical role in FMR. To provide posterior disclusion, the anterior guidance acts like an anterior control. The anterior guidance protects the posterior teeth from lateral and protrusive forces. The face bow transfer is required to connect the anterior guidance with the opening and closing axes. The arc of closure from the patient to the articulator is replicated using the face bow.

When replacing posterior teeth, three factors are paramount: achieving posterior disclusion, selecting an occlusion plane, and determining the type of occlusal scheme to be used. Christensen and D'Amico describe disclusion as separating opposing teeth during eccentric mandibular movements [15]. The posterior occlusion must have equal simultaneous contacts to avoid interfering with the TMJ or anterior guidance. Deflective occlusal interferences should be removed. A proper occlusion plane must allow the disclusion of all teeth on the balancing side when the mandible is moved laterally. An occlusion reconstruction should be made at the centric level, and the patient should be able to accept it at the neuromuscular level [16]. Among the contraindications of the Hobo twin-stage procedures are abnormal Wilson curves, abnormal Spee curves, and abnormally tilted/rotated teeth [17]. The vertical axis of the posterior teeth may be tilted abnormally. The standard effective cusp angle described in the twin-stage technique may not be applicable in these cases [18,19].

Monolithic zirconia ceramic crowns were chosen to ensure enough strength for the patient's treatment. Monolithic zirconia restorations manufactured exclusively through computer-aided design/computer-aided manufacturing (CAD/CAM) have significant advantages. These include higher flexural strength, conservative dental preparation, minimal wear on antagonists, pleasing aesthetics, requiring less laboratory time, and fewer visits. In addition, because they are monolithic, they lack the unwanted complication of chipping [20]. Therefore, high translucent monolithic zirconia (BruxZir® Esthetic Zirconia; Glidewell Dental, California, USA) crowns with natural translucency, beautiful shade matching, and superior strength were chosen for anterior maxillary teeth. The material has an average flexural strength of 900 MPa and was used with high-strength monolithic zirconia crowns (BruxZir® Full-Strength Zirconia; Glidewell Dental, California, USA) for the remaining teeth. This material provides better translucency and color than natural dentition, better shade consistency, higher flexural strength (˃1000 MPa), and prevents any shade change following occlusal correction [21-23].

## Conclusions

Various occlusal schemes have been proposed for FMR, including Pankey-Mann-Schulyer, Youdeli, and Hobo. The unique feature of Hobo's technique is that it provides a predictable posterior disclusion and anterior guidance that follows the condylar path. In addition, the cuspal angle and anterior guidance can be created precisely without concern about the remaining natural teeth since all teeth are restored in FMR. Furthermore, the amount of disocclusion can be reproduced precisely as programmed. Besides that, it's a simple procedure that doesn't need any special equipment, and it can also be done in one appointment.
